# Supplementary material for: A set of multi-entry identification keys to African frugivorous flies (Diptera, Tephritidae)
Source: Zookeys. 2014 Jul 24;(428):97–108. doi: 10.3897/zookeys.428.7366 (PMC4143993; doi:10.3897/zookeys.428.7366)
Supplement: Supplementary material 4 — Key to Capparimyia [file zookeys-428-097-s004.zip › SF4_ZooKeys_key to Capparimyia/lucid_player/help/help/multiple_states.htm]

Choosing states from multistate features


# Choosing states from multistate features

In Lucid3, all states in *Features
Available* are provided with checkboxes. Choose a state by clicking
on its checkbox. Entities that match the chosen state will remain in *Entities Remaining*; entities that do
not match will be moved to *Entities
Discarded*. The chosen state will also be written to the *Features
Chosen* list.

In Lucid keys, you may choose more than one state for any given feature.
For instance, if a key has a feature *Flower
colour* with states *white*,
*cream*, *yellow*,
*red*, and *blue*,
you may be unsure whether to choose the states *white*
or *cream* for your specimen. In
this case, choose both states. In normal identification mode, *Entities
Remaining* will list all entities with white *or*
cream flowers.
